# Gender Difference in Health-Care Expenditure: Evidence from India Human Development Survey

**DOI:** 10.1371/journal.pone.0158332

**Published:** 2016-07-08

**Authors:** Nandita Saikia, Moradhvaj, Jayanta Kumar Bora

**Affiliations:** 1 Centre for Study of Regional Development, Jawaharlal Nehru University, New Delhi, India; 2 Public Health Foundation of India, Gurgaon, India; University of Louisville, UNITED STATES

## Abstract

**Background:**

While the gender disparity in health and mortality in various stages of life in India is well documented, there is limited evidence on female disadvantage in health-care expenditure (HCE).

**Aims:**

Examine the gender difference in HCE in short-term and major morbidity in India, and understand the role of factors underlying the difference.

**Data and Methods:**

Using two rounds of nationally representative panel data—the India Human Development Survey (IHDS) 2004–2005 and 2011–2012 (IHDS I & II)—we calculate morbidity prevalence rate and mean HCE by gender, and examine the adjusted effect of gender on major morbidity-related HCE by using a two-part regression model. Further, we performed Oaxaca-Blinder decomposition of the gender gap in HCE in major morbidity to understand the contribution of demographic and socio-economic factors.

**Results:**

Health-care expenditure on females was systematically lower than on males across all demographic and socio-economic groups. Multivariate analysis confirms that female HCE is significantly lower than male HCE even after controlling demographic and socio-economic factors (β = -0.148, p = 0.000, CI:-0.206–0.091). For both short-term and major morbidity, a female disadvantage on HCE increased from IHDS I to IHDS II. For instance, the male–female gap in major morbidity related expenditure increased from INR 1298 to INR 4172. A decomposition analysis of gender gap in HCE demonstrates that about 48% of the gap is attributable to differences in demographic and socio-economic factors (endowment effect), whereas 50% of the gap is due to the differential effect of the determinants (coefficient effect).

**Interpretation:**

Indians spend less on female health care than on male health care. Most of the gender gap in HCE is not due to differential distribution of factors affecting HCE.

## Introduction

Gender disparity in health and mortality in India has been well documented in recent decades. The female advantage in life expectancy at birth (LEB) is a recent phenomenon in India, unlike in many parts of the world [1,2]. Also, the female advantage in overall LEB masks the disadvantage spread across ages: Indian females are still subject to feticide and excess mortality [3–5]. Further, even in the most recent periods, there is rampant gender discrimination in nutrition, immunization, treatment-seeking behavior, and living arrangements for both young and elderly populations [6–13].

A great deal of research has been conducted to understand gender discrimination in health-care utilization in India. Invariably, these studies document that men and women have unequal access to health care at various stages of the life cycle. For example, girl children are less immunized than boy children [9, 14]; have less access to hospital treatment; and are subject to fewer hospitalizations before death [15]. Also, girls’ access to health care depends heavily on the sex composition of siblings [9]. At old age, Indian women report worse self-rated health and disabilities, despite utilizing health-care facilities less [16, 17]. Also, untreated morbidity rates are higher among women than men, and a strong class gradient by gender is found for in-patient health care [18].

Is gender discrimination in health-care expenditure (HCE) in India as evident as in health outcomes? A survey of the literature suggests that gender difference in HCE has not received adequate attention in studies on India. A study of strategies used by Indian parents to finance HCE reveals that the chance of hospitalization among girls is lower than among boys; also, if boys are hospitalized, parents are substantially more likely to borrow, sell assets, or take help from friends to pay the expenses [19]. Parents consult qualified health professionals more often and sooner for boys than girls, and spend more money on boys than on girls [20]. A small study conducted in rural Uttar Pradesh showed that among households with male children, the average expenditure on health care during the neonatal period was nearly fourfold that of households with females [21]. Further, households with female newborns used cheaper public care providers, whereas households with male newborns preferred private providers (although unqualified) as these were perceived to deliver satisfactory care [21]. A study by Maharana and Ladusingh [22] reports that gender disparity among the elderly in health and food expenditure has been narrowing over time, and that the compositional shift of gender in households (from male-dominated to female-dominated) leads to the reduction of household expenditure on health and food. A longitudinal survey was conducted on rural cancer patients in a public tertiary health center in Odisha; a study based on it showed that expenditures on female adults were significantly less than those on male adults, and that about one-third of the difference can be attributed to gender discrimination [23]. However, while these studies document the nature and extent of gender discrimination in access to and expenditure on health care, these do not attempt to understand the socio-economic factors underlying the gender gap in HCE.

Using the most recent available nationally representative panel data, we examine whether the gender disparity in HCE continues over time. Unlike many earlier studies, we include both young and old populations, to understand gender discrimination in health overall. We then assess whether the gender gap in HCEs is explained by the differences in underlying demographic and socio-economic factors and, if so, to what extent demographic and socio-economic factors explain this gap.

## Data and Methods

### Data

We used two rounds of the longitudinal, nationally representative survey known as the India Human Development Survey (IHDS), conducted by researchers from the University of Maryland, USA and the National Council of Applied Economic Research (NCAER), New Delhi, India. The IHDS I (2004–2005) is a nationally representative, multi-topic survey of 41,554 households in 1,503 villages and 971 urban neighborhoods across India. The IHDS II (2011–2012) re-interviewed about 85% of these households (N = 42,152). Besides, new households were added to maintain a representative sample. Both the data sets are publicly available through the Inter-University Consortium for Political and Social Research (ICPSR). The IHDS conducted two one-hour interviews in each household, on topics such as caste, consumption, income, agriculture, education, health, employment, gender relations, etc. Children aged 8–11 years completed short reading, writing, and arithmetic tests. Additional village, school, and medical facility interviews are also available.

For both rounds of data, we used information available through the household questionnaire. Information on occurrence and duration of short-term morbidity, hospitalization, and related cost was collected for each member of household suffering from short-term morbidities. Similarly, information on diagnosis of major morbidities treatment, hospitalization, and related cost was collected through household questionnaire.

### Measures

If a household member had suffered fever, cough, or diarrhea in the 30 days preceding the survey, the household is described as having experienced a short-term morbidity. The question asked was “… Has anybody been ill with any of these (fever/cough/diarrhea) illness in [the] last 30 days?” Thus, short-term morbidity is a reported morbidity by the respondent on behalf of his family members. Treatment cost for short-term morbidity includes expenses for medical consultations, tests, medicines, transportation to the hospital, hospitalization, and surgery. Morbidities diagnosed by doctors are characterized as major. The question asked was “Has a doctor ever diagnosed any member of the household as having … cataracts, tuberculosis, hypertension, heart disease …?” The major morbidities listed in the questionnaires by the IHDs are cataracts, tuberculosis, high blood pressure, heart diseases, diabetes, leprosy, cancer, asthma, polio, paralysis, epilepsy, mental illness, sexually transmitted diseases, HIV/AIDS, and accidents. Further, IHDS collected information on type of treatment (public/private/traditional); hospitalization (yes/no); and cost due to doctor, hospital, surgery, test, medicines, and transportation to the hospital.

We analyzed the gender differential in HCE by

- demographic and socio-economic factors, such as age group (0–14, 15–59, 60+)
- place of residence (rural/urban)
- education of the individual (illiterate, up to primary, primary to secondary, higher secondary, and above)
- religion (Hindu, Muslim, others)
- caste (others, Other Backward Castes, Scheduled Castes, and Scheduled Tribes)
- wealth quintile (poorest, poor, middle, rich, richest)
- in-patient care (no, yes); and
- type of health facility (public, private, traditional healer).

### Statistical Methods

We conducted the analysis in STATA (version 13.1). We calculated the prevalence of short-term morbidity (major morbidity) by dividing the number of persons suffered from short-term morbidity (major morbidity) by the total number of persons in the sample. We did the chi-squared test to assess statistically significant difference in morbidity prevalence/ duration of sufferings by gender.

To examine the gender difference in health expenditure, we calculated mean health expenditure (MHE) by gender among various demographic and socio-economic subgroups. We adjusted the HCE figures to account for the inflation rates using the wholesale price index (as per base year 2004–2005 = 100; wholesale price index = 1.5)[24].

We carried out two-part model (2PM) in pooled data (IHDS I and IHDS II) to assess the adjusted effect of gender on major morbidity HCEs. The 2PM is one of the most popular models for analyzing HCE data [25]. Typically, the distribution of HCE has a mass of observations at one or more specific values—say, at zero—and is skewed on the right. Therefore, application of OLS linear regression models may yield biased and less precise estimates of means and marginal effect. The 2PM is a popular alternative in cases of probit (or logit) and OLS models. The first part (probit) estimates the probability that an individual will spend on health; the second part (generalized linear model (GLM), Gamma family, log link function) estimates the correlates of the positive level of expenditure [26]. Since medical expenditure is typically right-skewed, the log of the expenditure is modeled in GLM part. Also, two-part models are appropriate when the participation (visiting health facility) and consumption decisions (health expenditure) are chronologically sequential [27].

To examine the role of demographic and socio-economic factors in the gender gap in health expenditure, we used the Oaxaca-Blinder decomposition method [25,28,29]. The outcome variable is HCE in major morbidity. Therefore, the gap between mean outcome, Y ^male^ and Y ^female^, is equal to

$$\begin{array}{l} Y^{male}-Y^{female}\\ =\beta^{male}X^{male}-\beta^{female}X^{female}\\ =\beta^{female}X^{male}-\beta^{female}X^{female}+\beta^{male}X^{female}-\beta^{female}X^{female}+(\beta^{male}X^{male}-\beta^{female}X^{male}\\ -\beta^{male}X^{female}+\beta^{female}X^{female})\\ =\beta^{female}(X^{male}-X^{female})+X^{female}(\beta^{male}-\beta^{female})+(X^{male}-X^{female})(\beta^{male}-\beta^{female})\\ =\beta^{female}\Delta X+X^{female}\Delta \beta +\Delta X\Delta \beta \\ =E+C+CE \end{array}$$

In the above equation, x^male^ and x ^female^ are the vectors of explanatory variables evaluated at the means for male, and females, respectively. Thus, the gap between male and female HCEs is due to (a) a gap in endowments (or due to the distribution of Xs) (E) or (b) a gap in coefficients (C) or (c) a gap arising from interaction of endowment and coefficients (CE) (for a detailed description of this method, see O’Donnell et al. [25]).

## Results

### Sample description of the population suffering from short-term and major morbidity

Table 1 presents the sample description of people suffering from short-term and major morbidity in both rounds of the IHDS. Substantially more people suffer from short-term morbidity than major morbidities (24,059 against 11,776 in 2004–2005 and 34,526 against 20,129 in 2011–2012). In both rounds, the age composition of both types of morbidity is similar overall, but varies from one type of morbidity to another. In the case of major morbidities, a significant proportion of the sample is of adults aged 15–59 years (63.4% in 2004–2005 and 60.5% in 2011–2012). In both types of morbidity, the share of the elderly (aged 60+ years) increased from IHDS I to IHDS II. The share of males in short-term and major morbidities is slightly lower than that of females.

**Table 1:** Description of the sample by socio-economic, demographic and health care related factors, IHDS data 2004–2005 & 2011–2012.

	Short-term morbidity	Major Morbidity		
Factors	2004–05	2011–12	2004–05	2011–12
	(N = 24,059)	(N = 34,526)	(N = 11,776)	(N = 20,129)
**Age**				
0–14	49.5	40.8	6.4	5.2
15–59	42.7	48	63.4	60.5
60+	7.8	11.1	30.2	34.2
**Gender**				
Male	45.6	44.2	45.6	43.8
Female	54.4	55.8	54.4	56.2
**Place of residence**				
Rural	70.3	69.2	60	61.1
Urban	29.7	30.8	40	38.9
**Education level**				
Illiterate	51.1	45.7	41.4	40.3
Primary school	23	21.6	20.4	19.7
Secondary School	19.8	23.2	27.2	27.8
Higher secondary and above	6.2	9.5	11	12.2
**Marital status**				
Currently married	33.91	38.9	71.82	70.64
Single	60.62	53.88	12.19	11.33
Others	5.47	7.22	15.99	18.03
**Religion**				
Hindu	84.3	83.5	82.2	81.8
Muslim	12.5	14.1	12	13.6
Others	3.2	2.4	5.8	4.6
**Caste**				
General	37	27.3	43.6	34.9
OBC	34.3	42.5	34.3	42
SC/ST	28.8	30.2	22.2	23.1
**Wealth quintile**				
Poorest	15.8	19.1	9.1	11.3
Poorer	18.3	20.1	12.8	15
Middle	19.6	21.3	17.6	19.5
Richer	22.3	20.7	23	24.1
Richest	24	18.8	37.5	30.1
**Inpatient care**				
No	96.3	96.7	70.7	73.1
Yes	3.7	3.3	29.3	26.9
**Type of health facility**				
Public	26	25	33.3	33.9
Private	64.7	73	62.5	64.5
Traditional healer	9.3	2	4.2	1.6
Short term morbidity				
Fever	22.54	25.99	-	-
Cough	57.22	58.74	-	-
Diarrhea	20.24	15.27	-	-
**Major morbidity**				
Cataract	-	-	4.87	5.57
Tuberculosis	-	-	3.63	2.57
High BP	-	-	17.38	15.87
Heart disease	-	-	7.32	5.05
Diabetes	-	-	12.83	12.84
Asthma	-	-	9.61	7.33
Other long term disease	-	-	44.36	50.76

Table 1 also shows that a majority of the people belong to rural areas (over 60%); are illiterate or have completed primary school (varies between 60% and 70%); and are Hindu (over 80% and belong to any type of caste (OBC/SC/ST). A clear positive pattern is seen in the case of major morbidity by wealth quintile. As wealth quintile increases from poorest to richest, the share of the sample in major morbidity also increases.

### Gender differential in the prevalence of short-term and major morbidity

Table 2 shows the prevalence of short-term and major morbidity by gender in both rounds of the IHDS. The morbidity prevalence rate is consistently higher for females in both short-term morbidity (2004–2005: male 100.21 vs. female 124.10; and 2011–2012: male 154.05 vs. female 194.24) and major morbidity (2004–2005: male 49.11 vs. female 60.80; and 2011–2012: male 93.91 vs. female 119.86. A chi square test shows that the difference in prevalence rate by gender is statistically significant (p = 0.000).

**Table 2:** Prevalence rate of short-term and major morbidity by gender (prevalence = suffering from disease/total sample size)*1000.

	Short-term morbidity		Major morbidity	
	2004–2005	2011–2012	2004–2005	2011–2012
Male	100.21	154.05	49.11	93.91
Female	124.10	194.24	60.80	119.86

Note: The *p* value = 0.000 in chi square test assessing association between gender and morbidity prevalence for each type morbidity

### Gender difference in health care expenditure

**Fig 1:**
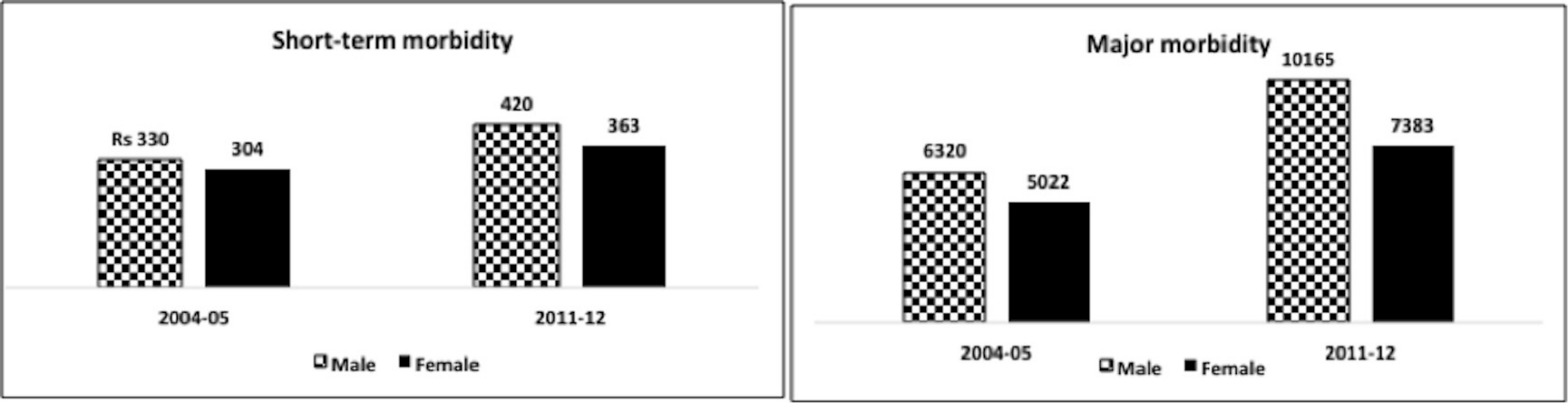
Mean health expenditure of short-term and major morbidity by gender expressed in Indian rupees (INR) 2004–2005 and 2011–2012 (at constant price).

The mean health expenditure expressed in Indian rupees (INR) by gender is shown in Fig 1 and Table 3. Mean health expenditure for women is always lower than that of men irrespective of type of morbidity and survey rounds. Table 3 also reports the gender difference in mean health expenditure (MHE) (= health expenditure of male—health expenditure of female) expressed in INR ($1 is approximately INR 65) by various factors. A negative value in this table indicates female HCE is higher than male HCE; a positive value indicates that the converse is true. Overall, a discernible difference in HCE was observed by gender. Male HCE was higher than that of female HCE irrespective of IHDS round and type of morbidity. After adjusting the inflation, male–female gap in short-term HCEs increased from INR 26 in 2004–2005 to INR 57 in 2011–2012. In major morbidity too, this gap increased from INR 1298 to INR 2781 in 2011–2012. A Chi square test shows that the increase in the difference in HCEs is statistically significant. Further, a vivid divide in HCEs by gender is visible across most demographic and socio-economic groups. An apparent class gradient is also revealed in the HCE on major morbidity in IHDS II. The gender gap in HCE among people in the richest wealth quintile is seven times that of people in the poorest wealth quintile (INR 704 vs. INR 5033).

**Table 3:** Gender difference in mean health expenditure expressed in Indian Rupees (INR)* by background factors, 2004–2005 and 2011–2012**.

	Short-term Morbidity		Major Morbidity	
Factors	2004–2005	2011–2012	2004–2005	2011–2012
	D1(Male-Female)	D2(Male-Female)	D1(Male-Female)	D2(Male-Female)
**Age**				
0–14	72	42	-635	97
15–59	16	103	1126	2977
60+	34	127	2211	2824
**Place of residence**				
Rural	17	51	1255	2902
Urban	53	72	1398	2571
**Education Level**				
Illiterate	43	17	847	1571
Up to Primary school	10	69	910	1577
Secondary School	15	82	1258	4247
Higher sec and A	6	139	362	1197
**Marital status**				
Currently married	18	128	1088	2562
Single	68	56	870	1684
Others	9	35	1248	1896
**Religion**				
Hindu	41	48	1084	2773
Muslim	-58	85	2108	2847
Others	-40	282	3466	2602
**Caste**				
General	-48	142	1822	2957
OBC	93	36	1219	2870
SC/ST	24	16	709	2448
**Wealth quintile**				
Poorest	41	45	477	704
Poorer	92	62	969	945
Middle	3	51	1271	1878
Richer	-24	70	714	2757
Richest	29	60	2295	5033
**Inpatient care**				
No	16	20	578	641
Yes	-101	500	2306	5564
**Type of health facility**				
Public	91	123	950	2055
Private	7	40	1628	3264
Traditional healer	-4	-23	-595	172
**Short term morbidity**				
Fever	17	76	-	-
Cough	24	43	-	-
Diarrhoea	43	67	-	-
**Major morbidity**				
Cataract	-	-	1416	-112
Tuberculosis	-	-	-2398	-4493
High BP	-	-	766	830
Heart disease	-	-	5052	11602
Diabetes	-	-	497	236
Asthma	-	-	-156	-117
Other long term disease	-	-	1610	3811
**Total**	**26**	**57**	**1298**	**2781**

* $1 = Indian Rupees 65

** Expenditures are at constant price based on WPI 2004–2005. Results are not subject to the choice of indices viz. CPI or WPI.

Table 4 demonstrates the results of the 2PM that examines the adjusted effect of gender on HCE in India in IHDS 2011–2012 data. The results of Probit model (zero expenditure versus any expenditure) shows that gender is not significantly associated with the outcome variable of spending any amount on HCE [ß = -0.030; p = 0.543; 95% CI (-0.126, 0.066)]. Likewise, age is not significantly associated with the outcome variable. After adjusting the effect of all background characteristics, it was found that the groups significantly associated with any health-care expenditure in major morbidity are urban resident, secondary school-educated, Muslims and people of OBCs, people in the middle and richer wealth quintiles, in-patient care seekers, and users of private health facilities.

**Table 4:** Two-part model estimates of factors affecting health care expenditure of major morbidity**, 2011–2012.

	Probit model (First part)				General linearized model (Second part)			
Factors	Coef.	P>z	95% CI		Coef.	P>z	95% CI	
**Age**								
0–14^®^								
15–59	0.076	0.619	-0.222	0.373	0.038	0.639	-0.120	0.196
60+	-0.041	0.795	-0.350	0.268	0.052	0.540	-0.115	0.219
**Gender**								
Male^®^								
Female	-0.030	0.543	-0.126	0.066	-0.148	0.000	-0.206	-0.091
**Place of residence**								
Rural^®^								
Urban	-0.200	0.000	-0.295	-0.106	-0.134	0.000	-0.191	-0.077
**Marital status**								
Currently married^®^								
Single	-0.038	0.709	-0.240	0.163	0.066	0.257	-0.048	0.180
Others	-0.129	0.033	-0.247	-0.011	-0.080	0.041	-0.158	-0.003
**Educational level**								
Illiterate^®^								
Primary School	-0.062	0.335	-0.188	0.064	-0.148	0.000	-0.221	-0.075
Secondary School	-0.169	0.006	-0.290	-0.048	-0.054	0.141	-0.125	0.018
Higher Secondary and above	-0.223	0.007	-0.387	-0.060	-0.035	0.481	-0.133	0.063
**Religion**								
Hindu^®^								
Muslim	0.280	0.000	0.123	0.437	0.013	0.746	-0.067	0.094
Others	-0.192	0.049	-0.384	0.000	0.072	0.272	-0.056	0.200
**Caste**								
General^®^								
OBC	-0.193	0.000	-0.300	-0.087	-0.092	0.004	-0.154	-0.030
SC/ST	-0.107	0.104	-0.237	0.022	-0.125	0.001	-0.202	-0.049
**Wealth quintile**								
Poorest^®^								
Poorer	0.127	0.110	-0.029	0.284	0.300	0.000	0.197	0.403
Middle	0.311	0.000	0.152	0.471	0.465	0.000	0.367	0.563
Richer	0.239	0.002	0.089	0.389	0.677	0.000	0.581	0.774
Richest	0.134	0.083	-0.017	0.286	1.119	0.000	1.020	1.218
**Inpatient care**								
No^®^								
Yes	0.390	0.000	0.269	0.511	1.234	0.000	1.172	1.296
**Type of health facility**								
Public^®^								
Private	0.870	0.000	0.774	0.967	0.187	0.000	0.131	0.243
Traditional healer	-0.375	0.000	-0.583	-0.167	-0.044	0.697	-0.267	0.178
**Type of major morbidity**								
Cataract								
Tuberculosis	0.382	0.013	0.082	0.683	1.271	0.000	1.053	1.488
High BP	0.385	0.000	0.204	0.567	0.242	0.001	0.097	0.387
Heart disease	0.421	0.001	0.176	0.666	1.271	0.000	1.099	1.443
Diabetes	0.327	0.001	0.143	0.512	0.743	0.000	0.594	0.891
Asthma	0.576	0.000	0.348	0.804	0.789	0.000	0.631	0.948
Other long term disease	0.624	0.000	0.456	0.792	0.973	0.000	0.839	1.107
^® reference category^		^ ^	^ ^	^ ^	^ ^	^ ^	^ ^	
**Number of observation** 19370								
**LR Chi2(21)**	670.5							
**Prob>chi2**	0.000							
**Pseudo R**^**2**^	0.160							

** Outcome variable: Total health care expenditure in major morbidity

The second part of 2PM shows the Generalized Linear Model regression results of modeling the factors of positive level expenditure in major morbidity. It is clear that the net effect of gender on the increasing level of HCE is negative and highly significant [ß = -0.148; p<0.001; 95% CI (-0.206, -0.091)]; that is, spending on major morbidity is less likely for females than for males. Similarly, spending on major morbidity is less likely for people living in urban areas, not currently married, illiterate, or of deprived castes. On the contrary, HCE increases with increase in wealth quintile, in-patient care seekers, and patients using private health facilities.

### Decomposition of gender in health care expenditure in major morbidity

Table 5 presents the results of Oaxaca decomposition to quantify the contribution of selected predictors in explaining the male-female difference in health care expenditure. The male-female difference in HCE is decomposed into three parts: the first part is known as endowment effect, which indicates the gap due to differences in the distribution of determinants between male and female; the second part is the coefficient effect, which indicates the gap due to the differences in the effect of determinants between the male and female; and the third is the interaction between both endowment effect and coefficient effect (interaction effect).

**Table 5:** Oaxaca decomposition:contribution by endowments, coefficients and interaction to Male-Female difference in health care expenditure, 2011–2012.

Mean prediction high (Male):	8.454			
Mean prediction low (Female):	8.21			
Raw differential (R) {Male-Female}:	0.244			
Due to endowments	0.119			
Due to coefficients	0.122			
Due to interaction	0.003			
% Explained by Endowment	48.77			
% Explained by coefficient	50.00			
% Explained by interaction	1.23			
Explanatory Variables	Endowments		Coefficients	
	Effect	% Contribution	Effect	% Contribution
Age	0.000	0.00	0.053	43.44
Place of residence	0.003	2.52	-0.032	-26.23
Educational level	-0.020	-16.81	0.033	27.05
Marital status	0.019	15.97	0.093	76.23
Religion	-0.001	-0.84	0.019	15.57
Caste	-0.002	-1.68	0.084	68.85
Wealth quintile	0.009	7.56	0.060	49.18
Inpatient care	0.077	64.71	0.018	14.75
Type of health facility	-0.006	-5.04	-0.036	-29.51
Morbidity type	0.017	14.29	-0.013	-10.66
Duration of stay in hospital	0.023	19.33	0.039	31.97
Constant	0.000	0.00	-0.194	-159.02
Total	0.119	100.00	0.122	100.00

The decomposition analysis reveals that about 48.77% of the male–female gap in HCE is explained by the differential distribution of demographic and socio-economic factors (Table 5). While education, religion, caste, and type of health facility contributed significantly towards reducing the male–female gap in HCE, place of residence, marital status, wealth index, in-patient care seeking, morbidity type and duration of stay in hospital contributed towards widening this gap. The contribution of in-patient care towards widening the gap was the highest (about 64.71%); it indicates that the distribution of in-patient care is more favorable to males than females. Next was duration of stay in hospital (19.33%). The highest contribution towards reducing the gap is by level of education (about 16.81%). About 50% of the difference was accounted by effects of determinants (coefficients). The positive contribution by marital status, caste, wealth quintile etc. indicates that effects of theses factors are responsible for wider gender gap in HCE. More precisely, the positive contribution of marital status (about 76.23% of total coefficient effects) suggests that effect of being married (or being widowed) is more favorable to male than females on HCE. There are a few offsetting factors such as place of residence, type of health facility and type of morbidity indicating the favorable effects of these variables to reduce the male-female gap in HCE. Since the contribution of interaction effect is marginal (about 1.23%), we did not present those results in detail.

## Discussion

The recognition of gender discrimination in health-care practices goes back to the early twentieth century.

*“There is no doubt that*, *as a rule*, *she [a girl] receives less attention than would be bestowed upon a son*. *She is less warmly clad*, … *…*. *She is probably not so well fed as a boy would be*, *and when ill*, *her parents are not likely to make the same strenuous efforts to ensure her recovery*” (1901 census, quoted in Miller [30]).

Yet, the recent literature focuses more on gender differentials in health outcomes, such as mortality and nutrition among children and, to a certain extent, among women in reproductive age groups. The importance of examining gender difference in the cost of chronic diseases grows tremendously due to the emergence of these diseases and its impact on disability status, particularly in adult and old ages. Using a recently available nationally representative data, this study attempted to answer three questions: Using a recently available nationally representative data, this study attempted to answer three questions: a) what is the nature and extent of gender discrimination in HCE in India in recent years? b) How much of this discrimination is due to the distribution of demographic and socio-economic factors and their effects? c) What are the factors of widening or reducing the gender gap in HCE?

This study puts forward two major findings. First, although both short-term and major morbidity are significantly higher among females than among males, average HCE is significantly lower among women. The disadvantage is more pronounced in the case of major morbidity since it incurs relatively higher costs. Further, except for a few cases of short-term morbidity in 2004–2005, the female disadvantage is prominent across all socio-economic categories in expenditure on both short-term and major morbidity. Contrary to common belief, gender discrimination in HCE is more severe in adult or old age than in young age. A strong gradient in gender discrimination is observed by household wealth quintile. These results hold true even after controlling the role of demographic, socio-economic, and health care-related factors.

The second important result is that less than half of the gender difference in HCE is due to male–female differences in demographic, socio-economic, and health care-related factors; the rest is due to the effects of these factors on HCE. Thus, the contribution by coefficients represents the genuine role of gender in HCE, i.e., less is spent on female health because of the notion that female health is not as important as male health.

The findings of this study are consistent with those of studies conducted in India and in some Asian countries [18, 20, 22, 23, 31, 32], but different from the results found from studies conducted in many developed and developing countries [33–35]. In general, women in developed countries are more aware about their health [36,37]; use more health-care facilities and preventive care [35,38]; and, therefore, spend more on their health [33, 39].

But in countries like India and China, where a complex web of poverty, social hierarchy, and deep-rooted patriarchal structure eliminates women’s health from the household priority list, women often invest more of their time in household activities or work that is not directly linked to economic outcomes [31, 40, 41]. Therefore, they may postpone meeting their own health needs to meet those of male family members directly involved in earning income [31, 42, 43] and prioritize the health of male members over their own [31, 44]. This is still an example of gender discrimination rather than gender equity since women’s indirect contribution to household income through household chores and care giving remains unaccounted. Secondly, in the absence of robust social protection schemes, poor families often mitigate health-related out-of-pocket expenditure by compromising on the health needs of female family members [15, 19, 44]. On the other hand, in non-poor families, women have less power to bargain on their needs due to lack of property ownership, lack of income earning means, lack of community support, and ongoing social norms and perceptions [45]. Thus, the gender differential in HCE in India is perhaps the result of powerful synergies between socio-economic status and patriarchal values.

This study has a few limitations. First, women respondents provided data on health-care expenditure incurred for all household members in the twelve months preceding the survey; therefore, there is a chance of recall bias of the expenditure data. However, this recall bias should be affected to both male and female health expenditure data and hence our results on gender difference might not be affected considerably. Secondly, by analyzing gender disparity in morbidity related expenditure, we are documenting only one part of discrimination that women may face in the process of health-seeking behavior. In reality, women may face sequential discrimination at the stage of health care i.e., in receiving out-patient care, in in-patient care, in major morbidity diagnosis and finally in arranging the health finance from various sources. This can be analyzed in future studies.

### Implications for Policy and Practice

Ensuring healthy lives for all and gender equality are two important sustainable development goals in the UN’s agenda [46]. To achieve these goals, it is crucial to identify the disadvantaged and excluded through the lens of gender. Previous studies show that although women in India live longer than men, they have poorer health outcomes, such as excess mortality in young ages, poor nutrition in young and adult ages, and higher rate of disability in old age [5, 17, 42]. The corollary of these findings is that women need better health-care support for India to reach gender equity in health. Unfortunately, the present analysis of HCE in India too reveals that gender equity in health care is a distant dream. To tackle gender-related discrimination in HCE and bring about equity, it is essential not only to introduce a social protection scheme but also to guarantee gender equitability in those schemes. Although the Rashtriya Swasthya Bima Yojana (RSBY), India’s social protection scheme, is a remarkable step towards addressing the health needs of disadvantaged groups, special emphasis should be given to foster health care utilization among women. At the same time, women empowerment and their involvement in decision-making powers is essential, since these are the vehicles of possible equity in health care services in India. Also, a deeper analysis of gender needs and gender barriers linking to social structure and health.
